# Evaluation of folate receptor 1 (*FOLR1*) mRNA expression, its specific promoter methylation and global DNA hypomethylation in type I and type II ovarian cancers

**DOI:** 10.1186/s12885-016-2637-y

**Published:** 2016-08-02

**Authors:** Sara Notaro, Daniel Reimer, Heidi Fiegl, Gabriel Schmid, Annamarie Wiedemair, Julia Rössler, Christian Marth, Alain Gustave Zeimet

**Affiliations:** 1 Department of Obstetrics and Gynecology, Medical University of Innsbruck, Anichstrasse 35, 6020 Innsbruck, Austria; 2 Department of Gynecology and Obstetrics, University of Brescia, P.zza Spedali Civili 1, 25123 Brescia, Italy

**Keywords:** Ovarian cancer, Folate receptor 1, FOLR1, FOLR1 promoter methylation, Global DNA hypomethylation, Type I, Type II, Platinum sensitivity

## Abstract

**Background:**

In this retrospective study we evaluated the respective correlations and clinical relevance of FOLR1 mRNA expression, FOLR1 promoter specific methylation and global DNA hypomethylation in type I and type II ovarian cancer.

**Methods:**

Two hundred fifty four ovarian cancers, 13 borderline tumours and 60 samples of healthy fallopian epithelium and normal ovarian epithelium were retrospectively analysed for FOLR1 expression with RT-PCR. FOLR1 DNA promoter methylation and global DNA hypomethylation (measured by means of LINE1 DNA hypomethylation) were evaluated with MethyLight technique.

**Results:**

No correlation between FOLR1 mRNA expression and its specific promoter DNA methylation was found neither in type I nor in type II cancers, however, high FOLR1 mRNA expression was found to be correlated with global DNA hypomethylation in type II cancers (*p* = 0.033). Strong FOLR1 mRNA expression was revealed for Grades 2-3, FIGO stages III-IV, residual disease > 0, and serous histotype. High FOLR1 expression was found to predict increased platinum sensitivity in type I cancers (odds ratio = 3.288; 1.256-10.75; *p* = 0.020). One-year survival analysis showed in type I cancers an independent better outcome for strong expression of FOLR1 in FIGO stage III and IV. For the entire follow up period no significant independent outcome for FOLR1 expression was revealed. In type I cancers LINE 1 DNA hypomethylation was found to exhibit a worse PFS and OS which were confirmed to be independent in multivariate COX regression model for both PFS (*p* = 0.026) and OS (*p* = 0.012).

**Conclusion:**

No correlations were found between FOLR1 expression and its specific promoter methylation, however, high FOLR1 mRNA expression was associated with DNA hypomethylation in type II cancers. FOLR1 mRNA expression did not prove to predict clinical outcome in type II cancers, although strong FOLR1 expression generally denotes ovarian cancers with highly aggressive phenotype. In type I cancers, however, strong FOLR1 expression has been found to be a reliable indicator of improved platinum responsiveness reflecting a transient better one-year follow up outcome in highly FOLR1 expressing type I cancers. An independent prognostic role of global DNA hypomethylation was demonstrated in type I tumours.

**Electronic supplementary material:**

The online version of this article (doi:10.1186/s12885-016-2637-y) contains supplementary material, which is available to authorized users.

## Background

Ovarian carcinomas account for the highest mortality among all gynecological cancers in the world [1]. Despite aggressive treatment including highly offensive surgical cytoreduction and multiagent chemotherapy, the prognosis of patients with advanced ovarian cancer remains unacceptably poor.

Folic acid is an essential component in DNA synthesis, replication and repair, protein synthesis and methylation reactions. This is especially true for rapidly dividing cells [2]. Three different mechanisms exist for cellular folate uptake: one via the membrane-associated Folate Receptor (FOLR) and the physiologically more important mechanism via the Reduced Folate Carrier (RFC). More recently, a third, the proton-coupled folate transporter, responsible exclusively for the intestinal uptake of folate, was identified [3, 4]. FOLR internalizes folates by means of receptor mediated endocytosis and RFC uses a bidirectional anion-exchange mechanism to transport folates into cytoplasm [5, 6]. Human FOLR is encoded by a family of genes whose homologous products are the FOLR types -α, -β and –γ.

Principally FOLR isoforms α and β are capable of transporting folate into cells, but generally the ubiquitously expressed RFC is exclusively used for this purpose by adult tissues [7]. In fact, most normal tissues virtually lack FOLR, and its physiologic importance appears to be confined to situations where the availability of folate is limited [8].

In contrast, the ability of FOLR1 (encodes for folate receptor α) to bind folate has been demonstrated in malignant tissue [9, 10]. Under such pathological circumstances FOLR1 may be overexpressed to increase folate uptake in order to cope with the augmented turnover of nucleic acid synthesis and reparation during accelerated cellular growth [11, 12]. FOLR1 was found to be strongly expressed in renal, pancreatic, endometrial carcinomas, squamous cervical cancer and ovarian cancer [13]. Furthermore, FOLR1 is reported to be expressed in the majority of non-mucinous epithelial ovarian cancers at levels 10- to 100-fold higher than its normal expression in the kidney and on lung and breast epithelial cells [14].

Recently, it was shown that FOLR1 can be exploited for specific delivery of drugs linked to folic acid into ovarian cancer cells. Proof of principle was provided with folate-desacetyl vinblastine monohydrazide (Vintafolide®) in the clinical PRECEDENT trial in ovarian cancer. Moreover, companion diagnostics with radio-labelled folate (Etarfolatide®) -based tumour imaging has been found to predict response to Vintafolide® [15].

Furthermore, folic acid is crucially involved in DNA methylation via metabolic cycling of methionine towards homocysteine. Epigenetic modifications of DNA through CpG site methylation are recognized to play a fundamental role in tumorigenesis. Two distinct DNA methylation abnormalities are observed in cancer. The first is a global genome-wide reduction of DNA methylation (global DNA hypomethylation) and the second is hypo- or hyper-methylation within the CpG islands within specific gene promoters. Promoter hypomethylation is believed to induce proto-oncogene activation, and global DNA hypomethylation is strongly related to chromosomal instability. Regional hypermethylation is strongly associated with transcriptional silencing of specific genes (e.g., tumour suppressor genes) [16]. A global decrease in the amount of cellular cytosine methylation is observed in many neoplastic tissues and is related to poor prognosis or clinical severity in several cancer types, including ovarian cancer [17].

The majority of global DNA hypomethylation occurs at repetitive elements, such as long interspersed nuclear elements (LINEs) [18]. Genome-wide DNA hypomethylation at LINE1 elements during tumorigenesis is presumed to contribute crucially to chromosomal instability [19].

Some authors [20–22] have proposed a potential role of epigenetic regulation in FOLR1 expression through methylation, but the evidence was not conclusive. Nevertheless, epigenetic changes can offer a plausible explanation for elevated FOLR1 expression in some tumours [23].

The purpose of the study presented here was to analyse the clinical relevance of FOLR1 mRNA expression and its possible influence on global DNA methylation status in ovarian cancer. Furthermore, we were interested in elucidating whether FOLR1 mRNA expression is mainly regulated by DNA methylation of its promoter.

In addition, the role of FOLR1 expression was assessed as predictive markers of platinum responsiveness. We were especially interested in conducting these analyses regarding the histologic subtypes and the ovarian cancer dualistic typology proposed by Kurman et al. [24, 25].

## Methods

### Patients and samples

Two hundred fifty four ovarian cancer samples were retrospectively analysed from 1989 to 2010 at the Medical University of Innsbruck. The study was performed in accordance with the principles of the Helsinki Declaration after approval by the local ethics committee.

Median age was 61.58 years. Median follow-up was 55 months (1-289 m). After surgery patients mostly received chemotherapy as a standard treatment. A minority of them (12 pts) underwent neoadjuvant treatment (at least three courses of chemotherapy followed by interval debulking surgery); these patients were excluded from the platinum response risk analysis. The majority received adjuvant chemotherapy platinum-based therapy. Staging was performed according to the International Federation of Gynecology and Obstetrics (FIGO) classification. All FIGO stages and all histotypes were included. The clinico-pathological characteristics are reported in Table 1. According to the dualistic model proposed by Kurman et al. we divided the cancers cohort into type I and type II [24, 25]. Clinical, pathological and follow-up data were stored in a database in accordance with hospital privacy rules. Tumour samples and clinical data were collected after written informed consent of patients. After primary treatment, all patients were monitored by our department at intervals increasing from three months to one year until death or the end of the study. Follow-up information was available for all patients. Overall survival was defined as the time from diagnosis to last follow-up or death and progression-free survival as the time from diagnosis to first recurrence. Median overall survival (OS) was 55.00 months (Q_25_-Q_75_ 22.00-96.25), and median progression-free survival (PFS) was 25.00 months (Q_25_-Q_75_ 10.00-74.25). To define sensitivity to platinum we calculated the time from the last course of chemotherapy as defined by Markman et al. [26]. Moreover, we analysed tissues from 60 healthy controls (23 tubes with fimbriae and 36 normal ovarian epithelial tissues) and from 13 ovarian borderline tumours. Median age of patients with borderline tumours was 54 years and 51 years for controls; we did not find any general association between age and FOLR1 mRNA levels (r_s_ = 0.044, *p* = 0.485). In borderline tumors there were nine patients with serous and four patients with mucinous tumours. Nine patients had FIGO stage I and four patients FIGO stage II borderline disease.

**Table 1:** Clinico-pathological features with univariate survival analysis in type I and type II ovarian cancers

		Type 1						Type 2					
		Median (years)	Q1-Q3	n.	%	*p*-value* PFS	*p*-value* OS	Median (years)	Q1-Q3	n.	%	*p*-value* PFS	*p*-value* OS
Age		59	49-73	122				62	55-71	132			
Histology	Serous			11	9.0 %					119	90.2 %		
	Endometrioid			57	46.7 %					13	9.8 %		
	Mucinous			41	33.6 %					0	0.0 %		
	Clear cell			13	10.7 %	0.076	0.123			0	0.0 %	*0.007*	*0.004*
FIGO stage	I			54	44.3 %					15	11.4 %		
	II			7	5.7 %					11	8.3 %		
	III			53	43.4 %					88	66.7 %		
	IV			8	6.6 %	*<0.0001*	*0.003*			18	13.6 %	*0.002*	*0.044*
Tumour grade	1			27	22.1 %					0	0.0 %		
	2			64	52.5 %					66	50.0 %		
	3			31	25.4 %	*0.008*	*0.015*			66	50.0 %	0.406	0.087
Residual disease	RD = 0			80	65.6 %					44	33.3 %		
	RD > 0			42	34.4 %	*<0.0001*	*<0.0001*			88	66.7 %	*<0.0001*	*<0.0001*
Chemotherapy	None			21	17.2 %					7	5.3 %		
	Adjuvant			100	81.9 %					111	84.1 %		
	Neoadjuvant			0	0.0 %					12	9.1 %		

Q = quartile; 1 = 25° 3 = 75°

### RNA extraction and reverse transcription

Tumour specimens were obtained immediately after surgery and brought to our pathologist. Part of the tissue was pulverized under cooling with liquid nitrogen and stored at -80 °C. Total cellular RNA extraction and reverse transcription of RNA were performed as recently described [27].

### *FOLR1* mRNA expression analysis

Primers and probes for the TATA box-binding protein (TBP; a component of the DNA-binding protein complex TFIID as an endogenous RNA control) were used as described by Bieche et al. [28]. Primers and probes for FOLR1 were determined using the computer program Primer Express (Life Technologies, Carlsbad, CA, USA). BLASTN searches were conducted to confirm the total gene specificity of the nucleotide sequences chosen for the primers and probes. To prevent amplification of contaminating genomic DNA, the probe was placed at the junction between two exons. FOLR1 forward primer: 5′-CTG GCT GGT GTT GGT AGA ACA G -3′; FOLR1 reverse-primer: 5′- AGG CCC CGA GGA CAA GTT-3′; FOLR1 TaqMan probe: 5′-CAT TCT TCC TCC AGG GTC GAC ACT GCT-3′-BHQ1.

PCR reactions were performed using an ABI Prism 7900HT Detection System (Applied Biosystems, Foster City, CA, USA) as recently described [27].

### DNA isolation and DNA methylation analysis

Genomic DNA from lyophilized, quick-frozen ovarian cancer specimens was isolated using the DNeasy tissue kit (Qiagen, Hilden, Germany). Bisulfite modification was performed using the EZ DNA Methylation-Gold Kit (Zymo Research, Orange, CA, USA) according to the manufacturer’s instructions. MethyLight analysis was done as described previously [29]. The PMR value (percentage of fully methylated reference) was calculated to determine the DNA methylation measurement.

Primers and probes for COL2A1 were described recently [29]. Primers and probes for FOLR1 were determined with the computer program Primer Express (Life Technologies, Carlsbad, CA, USA) to produce a 74-base-pair PCR amplicon (nucleotide positions 17662066-17662139 as defined by NCBI Reference Sequence NT_167190.2; gi|568815271) with a mean distance of -2.384 base pairs, to the transcription start site. The CpG island in the analysed gene was identified using the CpG island searcher (http://www.uscnorris.com/cpgislands/cpg.cgi) that screens for CpG islands that meet the criteria and algorithm described by Daiya Takai and Peter A. Jones [30]. The following primers were used for MethyLight PCR: FOLR1: Forward: 5′-CTC GAT CTC CTA ACC TCG TAA TCC-3′; Reverse: 5′-TAT GGT GGT TCG CGT TTG TAA TT-3′, TQM-probe: 6-FAM- 5′- CCC GCC TCG ACC TCC CAA AAT ACT T-3′-BHQ1;

LINE1 DNA-hypomethylation was analysed as recently described previously [29]; the levels of unmethylated repetitive elements were expressed as percent of unmethylated reference (PUMR) values and were calculated similarly to PMR (percentage of methylated reference) values except that bisulfite-converted human sperm DNA was used as an unmethylated reference for PUR determinations.

### Statistical analyses

To compare variables between two groups we used the Mann-Whitney U Test and between more than two groups Kruskal Wallis test. Analysis of survival was performed with Kaplan-Meier curves and log-rank test. The Cox regression analysis was used for multivariate survival analysis. To eliminate variables we applied a backward variable selection procedure. A *p* value of 0.1 was used to exclude variables; all other tests were performed using a 0.05 % level of significance. Risk was analysed with odds ratio and chi-square tests and Fisher's exact test to evaluate significant values. Correlations were performed with Spearman's rank test. All statistical analyses were performed using SPSS, version 22.

## Results

### Correlation between FOLR1 mRNA expression and FOLR1 promoter methylation

Neither in the whole cohort of ovarian cancers nor in the analysed type I and type II cancers a significant correlation between the expression of FOLR1 and its specific promoter methylation was revealed (Additional file 1: Figure S1).

### FOLR1 mRNA expression in ovarian cancer

**Fig. 1:**
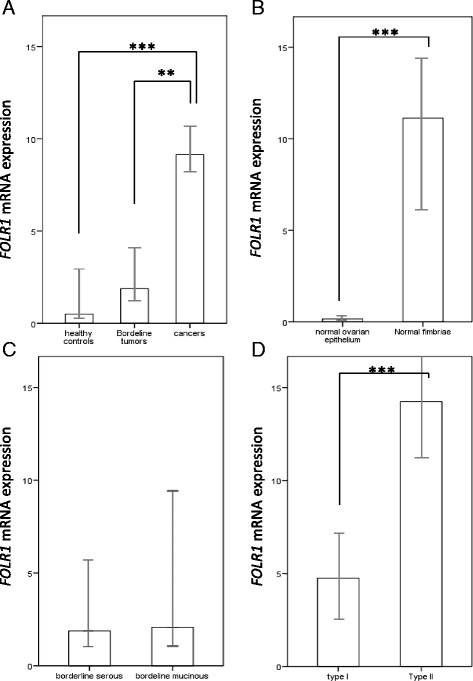
FOLR1 expression in healthy tissues, borderline tumours and cancers. FOLR1 mRNA expression (median values) in **a**) healthy controls (normal fimbriae and ovarian surface epithelium), borderline ovarian tumours (all histotypes) and ovarian cancers (all histotypes); **b**) in healthy fimbriae compared to healthy ovarian epithelium (*p* < 0.0001); **c**) in serous compared to mucinous borderline ovarian tumors (n.s.); **d**) in type I and type II ovarian cancers (*p* < 0.0001); Mann-Whitney test was applied (*P < 0.05, **P < 0.005, ***P < 0.0001). Units: arbitrary units normalized to TBP. Error bars: 95 % CI

In the unselected cohort of examined ovarian cancers, FOLR1 mRNA expression with a median value of 9.14 (arbitrary units normalized to TBP) was significantly stronger than in borderline tumours (median value: 1.88; *p* = 0.01) and in healthy controls (median value: 0.49; *p* < 0.0001) (Fig. 1a). In healthy control tissues, normal fimbriae of the fallopian tube (median value: 11.13) had stronger FOLR1 expression than normal ovarian epithelial tissue (median value: 0.16; *p* < 0.0001; Fig. 1b). In borderline tumors FOLR1 expression did not differ between serous and mucinous histotypes (Fig. 1c). In cancers FOLR1 was significantly stronger in type II (median value: 14.24) than in type I (median value: 4.75; *p* < 0.0001: Fig. 1d).

Two orders of magnitude can be distinguished regarding FOLR1 expression: one is the clustering of type I cancers together with borderline tumours and healthy ovarian epithelium and the second is the merging of type II cancers with healthy fallopian epithelium. The mean of mRNA expression of FOLR1 was 5.68 times higher in the second cluster compared to the first.

Table 2 shows differences in FOLR1 mRNA expression according to the classical clinico-pathological characteristics. Noteworthy is the significant higher levels of FOLR1 mRNA expression in high grade cancers, advanced stage, non-optimally debulked tumors and in platinum sensitive cancers. In serous histotypes FOLR1 expression was significantly higher than in non-serous cancer and in mucinous histotypes FOLR1 mRNA expression presented the lowest values (median value 2.99).

**Table 2:** Differences in FOLR1 mRNA expression and LINE1 DNA hypomethylation according to classical clinico-pathological characteristics

		FOLR1 mRNA expression^a^		LINE1-DNA hypomethylation^b^	
		Median value	*p*-value^1^	Median value	*p*-value^1^
Age (median value)	Lower	9.96		102.81	
	Higher	8.88	0.714	171.82	0.826
G1 VS G2-3	G1	2.55		88.40	
	G2-3	10.63	0.010**	138.34	0.436
FIGO stage	FIGO I, II	5.28		94.30	
	FIGO III, IV	11.94	<0.0001**	151.05	0.305
Residual disease	RD = 0	6.45		96.14	0.407
	RD > 0	12.64	<0.0001**	152.06	
Histology	Serous	14.04		118.29	
	Endometrioid	10.24		138.34	
	Clear cells	7.60			
	Mucinous	2.99	<0.0001**	104.06	0.533
	Serous	14.04		118.29	
	Non-serous	6.00	<0.0001**	125.62	0.602
	Mucinous	2.99		104.06	
	Non-mucinous	11.83		125.62	0.337
Platinum response	Refractory/resistant	6.82	0.049*	177.82	0.313
	Sensitive	11.68		117.71	
Type I/II	Type I	5.83		114.84	
	Type II	14.59	<0.0001**	134.67	0.345

^1^Mann Whitney Test. *Significant at the 0.05 level, **Significant at the 0.01 level, ^a^Arbitrary units normalized to TBP, ^b^PUMR values

### FOLR1 mRNA expression and clinical outcome

**Fig. 2:**
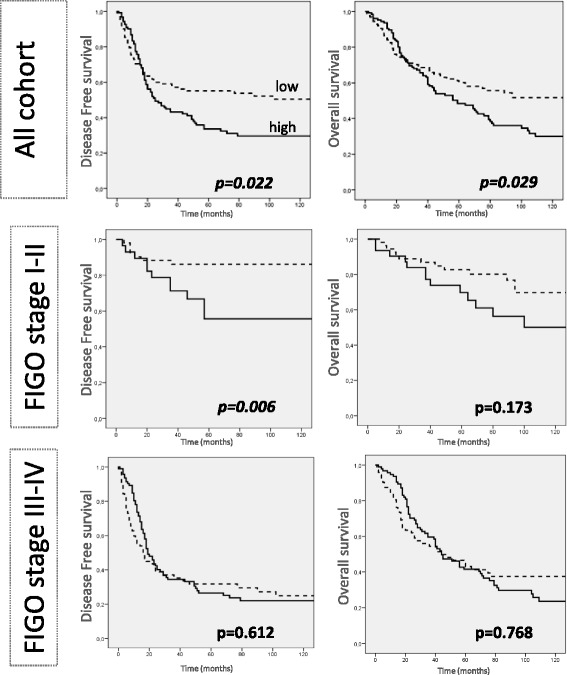
Survival curves (entire follow-up period) according to FOLR1 mRNA expression in the whole ovarian cancer cohort and stratified per FIGO stage. Kaplan-Mayer curves and log-rank test were applied. Cut off for FOLR1 expression: median values in the cancer cohort (9.14). Units: FOLR1 mRNA expression: arbitrary units normalized to TBP

In univariate survival analysis, when the cohort of cancers was dichotomized along the median value in high and low FOLR1 expression cancers, we revealed significantly reduced PFS (*p* = 0.022) and OS (*p* = 0.029) in cancers with strong FOLR1 mRNA expression. (Figure 2) This could not be confirmed in multivariate analysis (Additional file 2: Table S1). Univariate analysis, performed separately for type I and type II cancers, did not show significant results (Additional file 3: Figure S2). Figure 2 depicts survival curves stratified with regard to FIGO stage. Noteworthy is the significant worse PFS for high values of FOLR1 in FIGO stage I and II, which was not verified when type I and II cancers were analysed separately (Additional file 3: Figure S2). This was not confirmed in multivariate analysis.

**Fig. 3:**
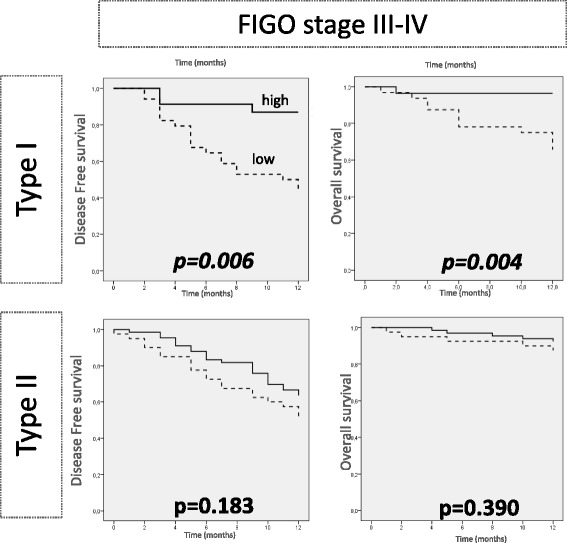
Survival curves limited to the first 12 months according to FOLR1 mRNA expression in type I and II cancers for FIGO stage III-IV. Kaplan-Mayer curves and log-rank test were applied. Cut off for FOLR1 expression: median values in the cancer cohort (9.14). Units: FOLR1 mRNA expression: arbitrary units normalized to TBP

It should be emphasised that during the first year follow-up strong expression of FOLR1 is significantly associated with better OS (*p* = 0.028). This tendency was also observed for PFS, but did not reach statistical significance (*p* = 0.226). When this one year analysis was performed separately in type I and type II cancers, no significant difference was found, but when stratification according to FIGO stage was performed, a small significant better PFS and OS for high levels of FOLR1 transcripts in type I cancers was revealed for FIGO stage III and IV (Fig. 3). This was confirmed in a multivariate COX regression analysis (Table 3).

**Table 3:** One-year follow-up univariate and multivariate survival analysis in FIGO stage III-IV for FOLR1 mRNA expression

			Univariate^a^								Multivariate^b^					
			PFS				OS				PFS			OS		
			Months (median)	CI 95 %	HR	*p* value	Months (median)	CI 95 %	HR	*p* value	HR	CI 95 %	*p* value	HR	CI 95 %	*p* value
Type I	FOLR1 mRNA expression^c^	Weak	8.56	7.06-10.06	0.246 [0.081-0.743]	0.006**	10.25	9.06-11.44	0.093 [0.012-0.717]	0.004**	0.38	0.16-0.87	0.023*	0.59	0.007-0.52	0.011*
		Strong	11.25	10.08-12.41			11.64	10.95-12.33								
	Grading	G1 VS G2-3									0.62	0.35-1.65	0.416	1.11	0.29-4.18	0.880
	Age	< o > median									0.76	0.36-1.58	0.461	1.46	0.69-3.05	0.317
	Residual disease	0 VS >0									2.04	0.71-5.83	0.183	1.33	0.37-4.29	0.661
	histology^d^										1.73	0.94-3.37	0.077	2.01	0.94-4.29	0.071
	FIGO stage	I-II VS III-IV									2.08	0.84-5.11	0.110	1.01	0.30-3.83	0.982
Type II	FOLR1 mRNA expression^c^	Weak	9.17	7.94-10.40	0.904 [0.577-1.414]	0.183	11.25	10.38-12.12	0.586 [0.170-2.023]	0.390						
		Strong	10.30	9.57-11.02			11.68	11.30-12.05								

Cut off for FOLR1 mRNA expression = median value in the cancer cohort (9.14) *Significant at the 0.05 level, **Significant at the 0.01 level ^a^log rank test, ^b^COX-regression analysis;

^c^Arbitrary units normalized to TBP. ^d^Serous, endometrioid, mucinous and clear cell. Multivariate analysis was performed if *p*-value in univariate analysis was > 0.1

The odds ratio to predict platinum refractority/resistance in case of high FOLR1 expression cancers, in the whole cohort, was 2.013 (1.086-3.731) *p* = 0.018. Moreover, performing the same analysis separately in type I and type II cancers, this odds ratio was increased to 3.288 (1.256-10.75; *p* = 0.020) in type I cancers. However, in type II cancers, results on platinum responsiveness were no longer significant (1.877, 0.833-4.226, *p* = 0.126).

### Correlation between FOLR1 mRNA expression and global DNA hypomethylation

As folate is an indirect supplier of methyl groups for general methylation processes in cells, one other aim of this work was to examine whether mRNA expression of FOLR1 was associated with the degree of global DNA methylation in ovarian cancer cells.

Analyses for global DNA hypomethylation were performed in the cancer cohort based on the methylation status of LINE1 as the principle surrogate marker.

Expression of FOLR1 was strongly associated with global DNA hypomethylation. This was shown by a significantly positive correlation between FOLR1 mRNA expression and unmethylated LINE1 repetitive elements measured by MethyLight PCR (LINE1-UR) (r_s_ = 0.298, *p* = 0.05). This relationship between global DNA hypomethylation and FOLR expression was observed in type II cancers (r_s_ = 0.390, *p* = 0.033), but not in type I cancers.

### Global DNA hypomethylation and clinical outcome

In Table 2 LINE1 hypomethylation values according to the main clinico-pathological parameters are shown. No significant differences were detected.

**Fig. 4:**
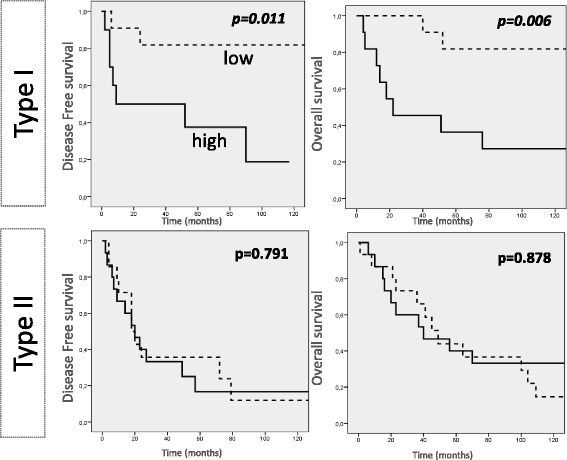
Kaplan Maier curves for LINE1 hypomethylation in type I and type II cancers. Kaplan-Mayer curves and log-rank test were applied. Cut off for LINE1 hypomethylation: median value in cancer cohort. Units: LINE1 hypomethylation: PUMR values, percent of unmethylated reference

Using the median value of LINE1 DNA hypomethylation in the cancer cohort (121.95) as the cut off value we distinguished two groups, patients with high and patients with low degree of LINE1 DNA hypomethylation in their tumors. In univariate analysis we disclosed that only in type I cancers both PFS (*p* = 0.011) and OS (*p* = 0.006) were reduced in high degree of LINE1 DNA hypomethylated cancers (Fig. 4). Independency of the latter results was confirmed in a multivariate COX-regression model both for PFS and OS (Table 4). However, no relevance on clinical outcome was revealed in type II cancers for global LINE1 hypomethylation.

**Table 4:** Univariate^b^ and multivariate survival^a^ analysis in type I and type II cancers for LINE1 DNA hypomethylation

			Univariate						Multivariate					
			PFS			OS			PFS			OS		
			Months	CI 95 %	*p* value	Months	CI 9 5%	*p* value	HR	CI 95 %	*p* value	HR	CI 95 %	*p* value
Type I	LINE1 DNA hypomethylation^c^	Low	208.9	154.8-262.8	0.011*	197.62	148.2-247.1	0.006**	6.13	1.24-30.22	0.026*	6.82	1.53-30.39	0.012*
		High	48.1	18.7-77.5		67.9	24.5-111.3							
	Grading	G1 VS G2-3							2.87	0.65-12.65	0.164	1.64	0.43-6.18	0.467
	FIGO stage	I-II VS III-IV							1.98	0.21-18.69	0.549	1.09	0.07-16.16	0.949
	Age	< o > median							0.61	0.11-3.27	0.566	3.84	1.09-13.52	0.036*
	Residual disease	0 VS >0							5.16	2.76-9.64	<0.0001**	21.6	1.23-377.66	0.035*
Type II	LINE1 DNA hypomethylation^c^	low	51.4	15.0-87.8	0.791	86.10	38.7-133.5	0,878						
		high	42.7	15.7-69.8		68.97	38.9-98.9							

*Significant at the 0.05 level, **Significant at the 0.01 level, ^a^log rank test, ^b^COX-regression analysis; ^c^PUMR values

## Discussion

The study presented here analysed FOLR1 mRNA expression, its promoter DNA methylation and global DNA hypomethylation in ovarian cancer and focused especially on differences between type I and type II cancers. To the best of our knowledge no specific investigations of this issue have been performed to date in ovarian cancer, even though, the study presented here certainly has some limits such as its intrinsic retrospective design and consequently the lack of statistical balance in the analysed subgroups that inevitably leads to structural bias.

We were unable to evidence a relevant relationship between FOLR1 mRNA expression and the specific FOLR1 promoter methylation. This was also true when type I and type II cancers were evaluated separately. There is only a small CpG-island upstream of the transcription stat site of FOLR1 (-2552 to -2331 bp; 222 bp region containing 11 CpGs). 7 of these 11 CpG sites are covered by our MethyLight PCR reaction. Recently Stewart et al. [31] analysed the impact of a decitabine treatment, a DNA demethylating agent, on the expression of RhoA, FOLR1 and RFC1 in 31 tumour patients. They observed that there was no increase in FOLR1 protein expression after a treatment with decitabine. Therefore, taken all these together, it seems that DNA methylation does not play a relevant role in the regulation of FOLR1 expression.

The dualistic typology described by Kurman et al. [24, 25] is based on the different origin and pathogenesis of both types of ovarian cancers Type I tumours are typically more indolent and frequently diagnosed in stage I. They are characterized by specific DNA mutations that perturb signalling pathways [24, 32], but exhibit relative genetic stability and are thought to develop from well-established precursors, the borderline tumours [24]. The more aggressive type II tumours (largely represented by high-grade serous cancers) are typically present at an advanced stage, with higher fatality and are thought to originate from tubal epithelium via serous tubal intraepithelial carcinoma (STICs) [24, 25, 33]. We noticed that FOLR1 mRNA expression is significantly stronger in both the tubal healthy epithelium and the type II cancers than in normal ovarian epithelium together with borderline tumours and type I cancers. These observations suggest that mRNA expression of FOLR1 in cancers is maintained from the cells of origin and is cancer type-specific.

Moreover, we are able to underscore the hypothesis established by others [34, 35], namely that strong FOLR1 mRNA expression appears to be related to a more aggressive cancer phenotype, as shown by the association with higher-graded tumours, more residual disease after primary debulking surgery, and the association with advanced FIGO stages.

Accordingly, univariate survival analyses for the entire cohort of patients revealed poor clinical outcome in terms of PFS and OS when FOLR1 mRNA expression was up-regulated, but this was not verified when type I and type II cancers were assessed separately. Similarly, RT-PCR based results reported by the by Siu et al. [35] did not reveal prognostic relevance for FOLR1 expression. However, the study of Chen et al. [34] reported an independent worse DFS and OS for high levels of FOLR1 in serous ovarian cancers in a RT-PCR based analysis.

Type I cancers are known for their poor constitutive responsiveness to chemotherapy including platinum agents and, as already mentioned, FOLR1 expression has been found to be significantly weaker in type I and type II cancers. Of special note, however is, that a subgroup of type I cancers showing FOLR1 expression above median value 9.14 exhibited a far stronger sensitivity to platinum treatment than did the large majority of type I tumours. Even if we used the classical arbitrary threshold of the six-month platinum-free interval to assign platinum sensitivity as defined by Markman et al. [26], which may be less appropriate in type I cancers due to their low constitutive proliferation, it appears that this subgroup of relatively “strong” FOLR1 mRNA-expressing type I cancers has a more aggressive phenotype that is closer to that of type II cancers and may thus be more susceptible to platinum agents. Nonetheless, we were not able to show this relationship in type II ovarian cancers, which show *a priori* stronger FOLR1 mRNA expression. Contrary to our findings, others reported FOLR1 expression to be predictive for poor platinum response in serous cancers [34], or were unable to detect a relationship between FOLR1 expression and chemosensitivity [35].

Although, FOLR1 appeared to be related to the aggressiveness of the disease, a peculiar transitory inverted effect in the one-year survival analysis was detected. In FIGO stage III and IV type I cancers showed independent better PFS and OS for high levels of FOLR1 mRNA expression. This small and transitory PFS and OS effects might be consistent with the association of FOLR1 expression with platinum sensitivity in type I cancers.

Even though Immunohistochemistry evaluation is not directly comparable with results obtained with RT-PCR, a similar transient inverted time limited effect was already described by Köbel et al. [36]. These authors found, for high values of FOLR1 mRNA expression, a better independent OS for serous cancers in the first two years follow-up.

A further important aspect of the determination of FOLR1 expression is that FOLR1 may potentially represent a molecule of major interest as a target for folate-conjugates using highly efficient drugs and the principal efficacy of this treatment option has already been evaluated together with a highly predictive companion diagnostic in clinical trials [37]. Probably those patients with type I cancers and high expression of FOLR1 may benefit from these treatments.

Another goal of our work was to investigate whether FOLR1 expression is associated with the degree of global DNA hypomethylation, which is considered to be a sign of unfavourable prognosis in ovarian cancer [38]. It has been shown that folate depletion, which potentially causes upregulation in FOLR1 expression [22, 23], induces global DNA hypomethylation [39]. In fact, our results for the first time provide evidence that FOLR1 expression could be crucially involved in the modulation of global DNA methylation especially in type II ovarian cancer and possibly also in other malignancies.

LINE1 DNA was used as a direct marker for global DNA hypomethylation. Our cohort of type I ovarian cancers showed an independent worse PFS and OS for high degree of LINE1 hypomethylation. This was in agreement with our previous study [29] where poor PFS and OS were found in mucinous ovarian cancers. However, in the present study we demonstrate a prognostic value of LINE1 DNA hypomethylation not only in a specific histotype but also in a well genetically and clinically characterized group of ovarian cancers, mainly the type I cancers. Certainly, in depth investigations are requested to confirm These Data to elucidate the underlined molecular mechanism connecting FOLR1 expression to the global methylation status of ovarian cancers.

## Conclusion

We conclude that according to FOLR1 expression our data evidenced a completely different behaviour between type I and type II ovarian cancers in terms of clinical outcome and platinum sensitivity. Thus, our findings on FOLR1 add further considerable hints to the notion that both types of ovarian cancer should be considered as completely different clusters of tumors. Our results prompt us to speculate that in type I cancers, which are generally regarded to be chemoresistant, the subset of strong FOLR1-expressing tumours are those, which may benefit from platinum-based chemotherapy and may furthermore have a special advantage from treatment with folate-conjugates using platinum or other cytotoxic agents.

## Abbreviations

FOLR1, folate receptor 1; OS, overall survival; PFS, progression-free survival; IHC, immunohistochemistry; RT-PCR, real-time PCR; CI, Confidence Interval; HR, hazard ratio; OR, odds ratio; LINEs, long interspersed nuclear elements; Chr. 1 Sat2, chromosome 1, satellite 2 DNA sequences; Chr. 1 Satα, chromosome 1, satellite α DNA sequences; PMR value, PUMR values percentage of fully methylated reference; percent of unmethylated reference; TBP, TATA box-binding protein

## Additional files

Additional file 1: Figure S1. Associations between FOLR1 mRNA expression and FOLR1 promoter methylation. Scattered plot with Spearman‘s Rho correlations between FOLR1 mRNA expression and FOLR1 promoter methylation in A) the whole cohort of ovarian cancers; B) type I cancers and C) type II cancers. On the graphs are reported Spearman index and p-values for each group. Units: § arbitrary units normalized to TBP. §§ PMR values. (PPTX 86.4 kb) Additional file 2: Table S1. Multivariate survival analysis for FOLR1 expression in the entire cohort. Cut-off for FOLR1 expression = Median value in cancers. (XLSX 11 kb) Additional file 3: Figure S2. Survival curves (entire follow-up period) according to FOLR1 mRNA expression for type I and type II cancers and stratified per FIGO stage. Kaplan-Mayer curves and log-rank test were applied. Cut off for FOLR1 expression: median values in the cancer cohort (9.14). Units: FOLR1 mRNA expression: arbitrary units normalized to TBP. (PPTX 172 kb)
