# Supplementary figures and images for: Evaluation of folate receptor 1 (FOLR1) mRNA expression, its specific promoter methylation and global DNA hypomethylation in type I and type II ovarian cancers
Source: BMC Cancer. 2016 Aug 2;16:589. doi: 10.1186/s12885-016-2637-y (PMC4971744; doi:10.1186/s12885-016-2637-y)

## Slide 1
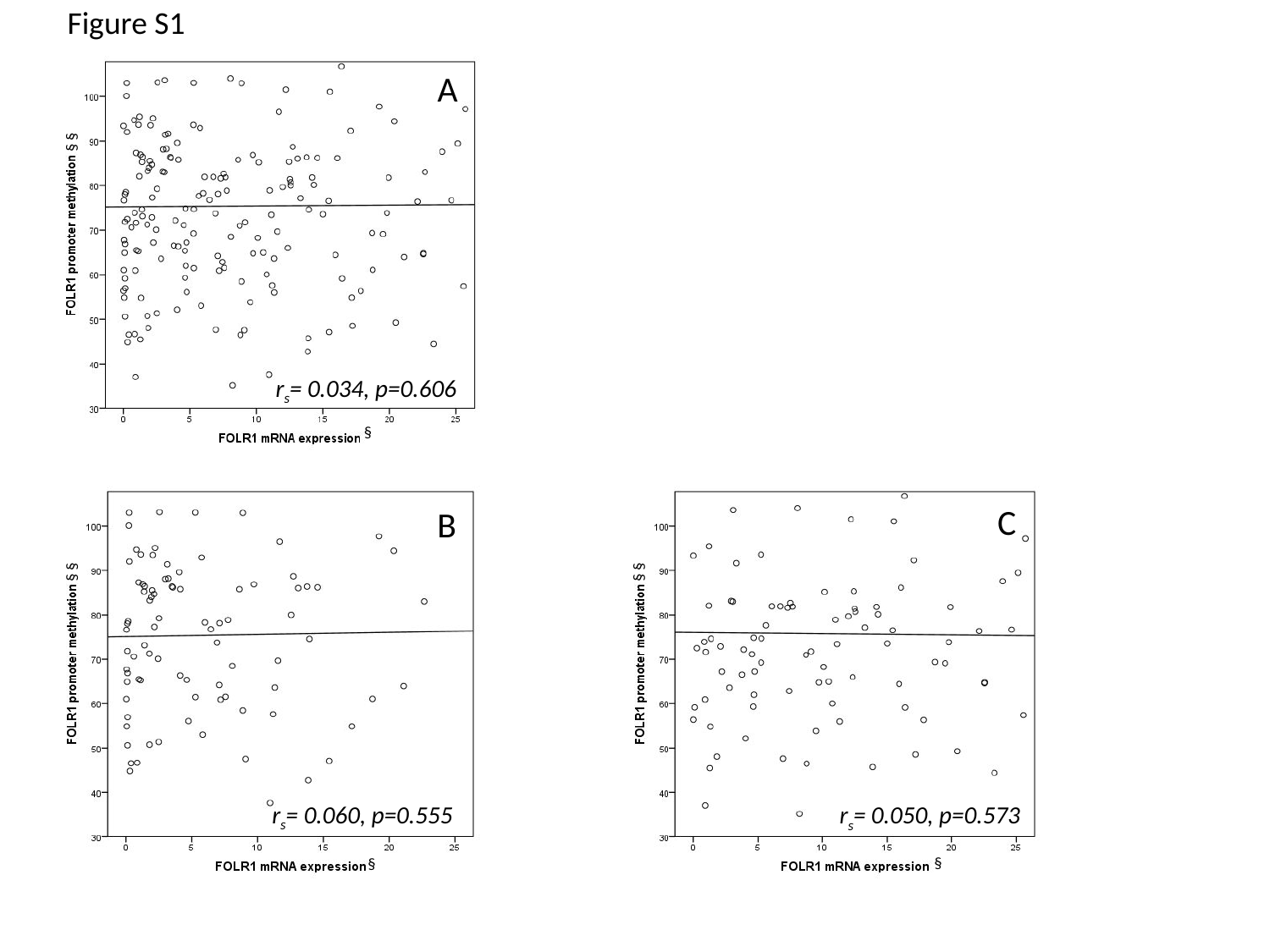

Figure S1
A
§ §
rs= 0.034, p=0.606
§
C
B
§ §
§ §
rs= 0.060, p=0.555
rs= 0.050, p=0.573
§
§

Supplement: Additional file 1: Figure S1. — Associations between FOLR1 mRNA expression and FOLR1 promoter methylation. Scattered plot with Spearman‘s Rho correlations between FOLR1 mRNA expression and FOLR1 promoter methylation in A) the whole cohort of ovarian cancers; B) type I cancers and C) type II cancers. On the graphs are reported Spearman index and p-values for each group. Units: § arbitrary units normalized to TBP. §§ PMR values. (PPTX 86.4 kb) [file 12885_2016_2637_MOESM1_ESM.pptx]
