# Supplementary material for: Evaluation of folate receptor 1 (FOLR1) mRNA expression, its specific promoter methylation and global DNA hypomethylation in type I and type II ovarian cancers
Source: BMC Cancer. 2016 Aug 2;16:589. doi: 10.1186/s12885-016-2637-y (PMC4971744; doi:10.1186/s12885-016-2637-y)
Supplement: Additional file 3: Figure S2. — Survival curves (entire follow-up period) according to FOLR1 mRNA expression for type I and type II cancers and stratified per FIGO stage. Kaplan-Mayer curves and log-rank test were applied. Cut off for FOLR1 expression: median values in the cancer cohort (9.14). Units: FOLR1 mRNA expression: arbitrary units normalized to TBP. (PPTX 172 kb) [file 12885_2016_2637_MOESM3_ESM.pptx]

## Slide 1
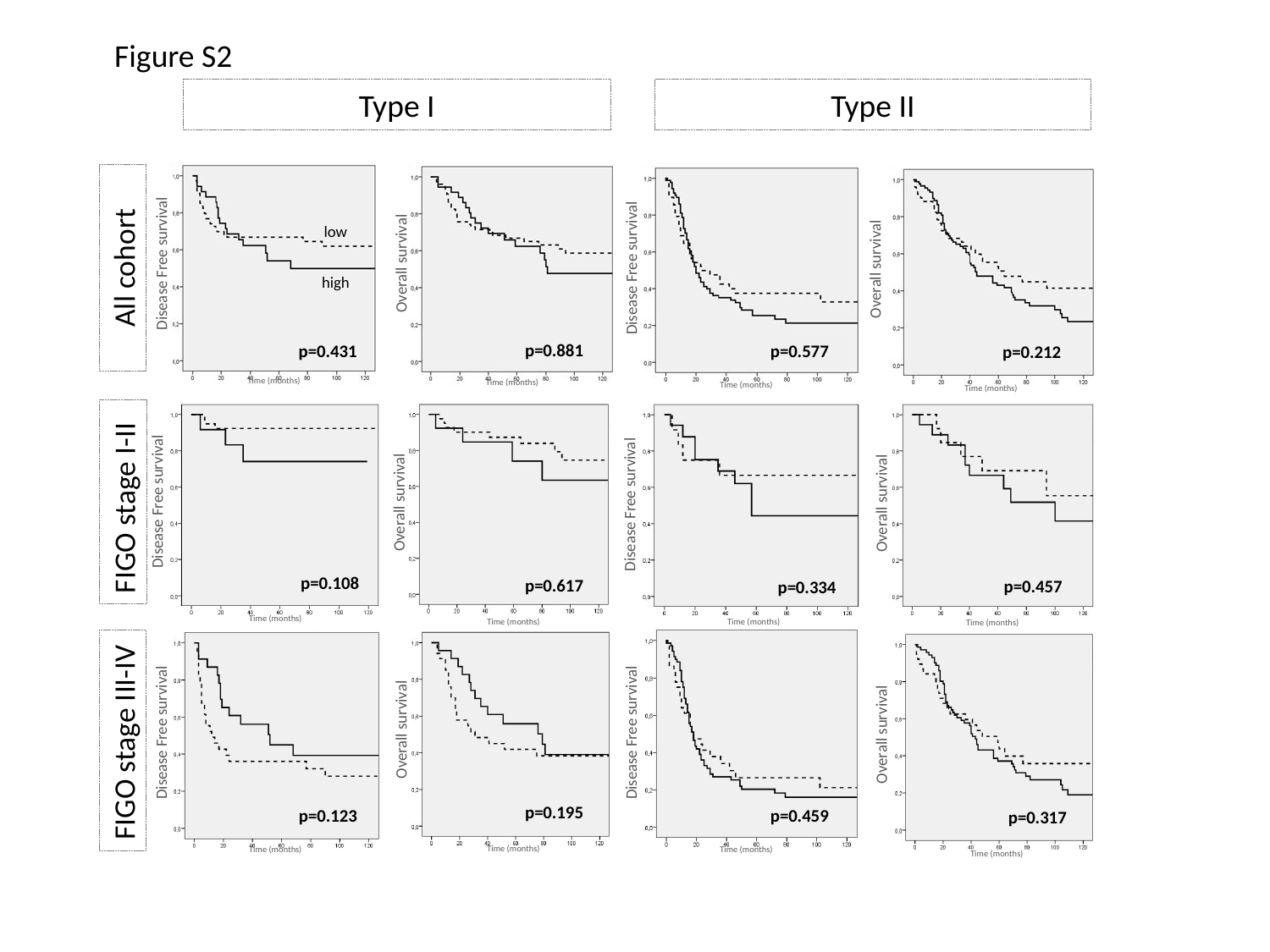

Figure S2
Type I
Type II
Disease Free survival
p=0.577
Time (months)
Overall survival
p=0.212
Time (months)
Disease Free survival
Overall survival
p=0.881
p=0.431
Time (months)
Time (months)
Overall survival
p=0.617
Time (months)
Overall survival
p=0.457
Time (months)
Disease Free survival
p=0.334
Time (months)
Disease Free survival
p=0.108
Time (months)
Overall survival
p=0.195
Time (months)
Disease Free survival
p=0.123
Time (months)
Disease Free survival
p=0.459
Time (months)
Overall survival
p=0.317
Time (months)
low
All cohort
high
FIGO stage I-II
FIGO stage III-IV
